# Numerical Well Testing Interpretation Model and Applications in Crossflow Double-Layer Reservoirs by Polymer Flooding

**DOI:** 10.1155/2014/890874

**Published:** 2014-06-26

**Authors:** Haiyang Yu, Hui Guo, Youwei He, Hainan Xu, Lei Li, Tiantian Zhang, Bo Xian, Song Du, Shiqing Cheng

**Affiliations:** ^1^MOE Key Laboratory of Petroleum Engineering, China University of Petroleum, Beijing 102249, China; ^2^Department of Petroleum Engineering, University of Texas at Austin, Austin, TX 78712, USA; ^3^Research Institute of Exploration and Development, PetroChina, Korla 841000, China; ^4^Department of Petroleum Engineering, Texas A&M University, College Station, TX 77843, USA

## Abstract

This work presents numerical well testing interpretation model and analysis techniques to evaluate formation by using pressure transient data acquired with logging tools in crossflow double-layer reservoirs by polymer flooding. A well testing model is established based on rheology experiments and by considering shear, diffusion, convection, inaccessible pore volume (IPV), permeability reduction, wellbore storage effect, and skin factors. The type curves were then developed based on this model, and parameter sensitivity is analyzed. Our research shows that the type curves have five segments with different flow status: (I) wellbore storage section, (II) intermediate flow section (transient section), (III) mid-radial flow section, (IV) crossflow section (from low permeability layer to high permeability layer), and (V) systematic radial flow section. The polymer flooding field tests prove that our model can accurately determine formation parameters in crossflow double-layer reservoirs by polymer flooding. Moreover, formation damage caused by polymer flooding can also be evaluated by comparison of the interpreted permeability with initial layered permeability before polymer flooding. Comparison of the analysis of numerical solution based on flow mechanism with observed polymer flooding field test data highlights the potential for the application of this interpretation method in formation evaluation and enhanced oil recovery (EOR).

## 1. Introduction

Over the past several decades, many EOR methods were researched in laboratories and oilfields to improve oil recovery, for example, polymer flooding [1], surfactant flooding [2], alkali-surfactant-polymer (ASP) flooding [3], nanoparticles [4, 5], low salinity water flooding [6], and CO_2_ [7, 8]. However, polymer flooding is most commonly applied in oilfields, especially hydrolyzed polyacrylamide (HPAM) polymer flooding because of its low cost and high efficiency [9]. The oil recovery of polymer flooding is enhanced mainly by increasing sweep efficiency [10].

Conventional pressure transient test has historically been the main application of permeability and skin estimation in oilfields, by using a pressure gauge positioned at a fixed depth in a well. The pressure test of multilayered reservoir was studied from the 1960s; however, the research on the individual production of multilayered reservoir was not carried out, due to the restriction of testing tools and technology. A percolation model of multilayered reservoir was derived in 1961, and the wellbore pressure and production of individual layers were also deduced [11]. This model considered that the interlayer had different parameters but neglected the wellbore storage effect. In 1978, a new model was further developed to get the wellbore pressure solution in real space for multilayered reservoir by using Stehfest algorithm [12]. It took the wellbore storage and skin factor into account, whereas it ignored the crossflow of wellbore pressure response. From the 1980s to 1990s, many researchers interpreted well testing data by analysis of measured wellbore pressure and stratified flow rate. With the help of multilayer testing techniques, the expression of pressure solution was established through the relationship between wellbore pressure and stratified flow rate of multilayered reservoir [13, 14]. The well testing model of crossflow double-layer reservoir was put forward in 1985 [15], which was further investigated by theoretical study of flow mechanics [16]. However, the type curves of crossflow double-layer reservoirs were not established. The problem of interlayered crossflow in a stratified reservoir was mathematically simplified by employing a semipermeable wall model [17]. Based on the former research, the dynamic model and exact solution of bottom hole pressure were proposed. Most researches on well testing and fluid percolation in double-layer reservoirs were based on analysis method to get the analytic solution of bottom hole pressure (BHP). In recent years, the numerical methods were employed to study well testing problems of multilayered reservoir with the help of rapid development of computer technology [18–21].

HPAM polymer solution is one kind of non-Newtonian fluids, and its viscosity is a significant parameter used to establish well testing interpretation model for polymer flooding. Many researches on the rheological behavior of polymer solution simply consider polymer as power law fluid and using constant power exponent model to represent the percolation of polymer solution in reservoirs [22–25], which is unable to meet the actual demands of our oilfields. For crossflow double-layer reservoirs by polymer flooding, there exist not only shear effect and viscoelastic effect but also physic-chemical interaction during polymer solution percolating in porous medium, whereas the constant power exponent viscosity model ignores diffusion and convection of polymer during transport in porous medium. Meanwhile, the adsorption of polymers in the porous medium results in IPV [1, 6, 26, 27] and permeability reduction [28–31], which also needs to be taken into account.

At present, well testing models and techniques in double-layer reservoirs by water flooding become mature, and commercial software can be used for reservoir evaluation; however, well testing models and interpretation methods in reservoirs with crossflow by polymer flooding are still less. The purpose of this study is to establish well testing interpretation method that can be applied in crossflow double-layer reservoir by polymer flooding, by considering shear, diffusion, convection, IPV, permeability reduction, wellbore storage effect, and skin factors. Moreover, field test data are further interpreted by this method for formation evaluation and EOR.

## 2. Polymer Rheology in Porous Medium

### 2.1. Materials

A proprietary HPAM used for polymer flooding was supplied by CNPC. The degree of hydrolysis is 25% and molecular weight of HPAM is 4050. The formation brines used in this study were prepared with salts of NaCl, MgCl_2_, CaCl_2_, and Na_2_SO_4_, and the synthetic brine composition is listed in Table 1. The total salinity, the sum of the ionic concentration, is 4.3 wt% (43000 ppm or 42.95 g/L).

### 2.2. Rheological Model

Polymer solution was assumed to behave as pseudoplastic non-Newtonian fluid. As discussed above, the power law model [32] or Carreau model [33] cannot accurately illustrate rheological behavior of the polymer used in our case. In this study, polymer shear-thinning behavior was simulated by use of Meter equation [34]:

(1) $$\begin{array}{c} \mu_{p}=\mu_{\infty }+\frac{\mu_{p}^{0}-\mu_{\infty }}{1+{\left(\gamma /\gamma_{1/2}\right)}^{P_{a}-1}\;}=\left(\mu_{w}+\frac{\mu_{p}^{0}-\mu_{w}}{1+{\left(\gamma /\gamma_{1/2}\right)}^{P_{a}-1}}\right), \end{array}$$

where *μ*_*p*_ is apparent viscosity of polymer solution; *μ*_*∞*_ is viscosity of polymer solution at infinite shear rate, which is simplified as brine viscosity (*μ*_*w*_) and satisfied the accuracy in this study since polymer concentration is relatively low and its viscosity at infinite shear rate is pretty close to brine viscosity; *γ*_1/2_ is the shear rate at which apparent viscosity is the average of *μ*_*∞*_ and *μ*_*p*_^0^; *γ* is the effective shear rate; *P*_*a*_ is a fitting parameter (usually 1.0 < *P*_*a*_ < 1.8); *μ*_*p*_^0^ is the viscosity at very low shear rate, which is calculated by modified Flory-Huggins equation [35]:

(2) $$\begin{array}{c} \mu_{p}^{0}=\mu_{w}\left[1+\left(A_{1}C_{p}+{A_{2}C}_{p}^{2}+{A_{3}C}_{p}^{3}\right)C_{\text{SEP}}^{\text{SP}}\right], \end{array}$$

where *A*_1_, *A*_2_, and *A*_3_ are fitting parameters obtained from matching experimental data; *C*_*p*_ is polymer concentration; *C*_SEP_^SP^ represents the effect of salinity and hardness on polymer viscosity.

**Figure 1:**
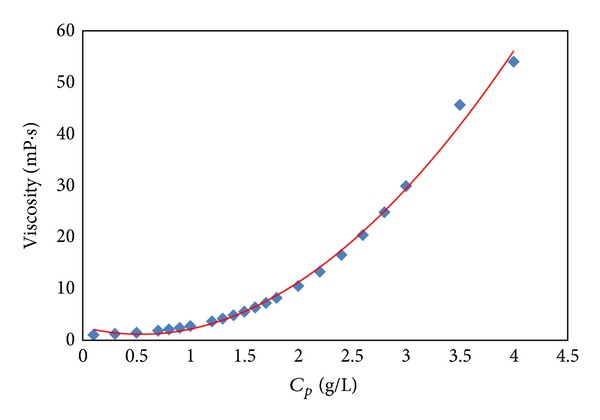
Relationship between polymer viscosity (*μ*_*p*_^0^) and polymer concentration (*C*_*p*_) at 75°C under 0.01 s^−1^ shear rate.

Since temperature significantly affects rheological behavior of polymer and the effect of pressure on polymer viscosity is negligible compared with temperature, the polymer solutions were prepared by mechanical stirring at 75°C to simulate reservoir temperature. The tested polymer concentrations range from 100 mg/L (0.1 g/L or 0.01 wt%) to 4000 mg/L (the polymer concentrations in our field tests are between 1600 mg/L and 2500 mg/L). The polymer rheological measurement was carried out by Haake RS6000 rheometer made in Germany. The viscosity of polymer solutions with different concentrations was measured at 75°C to get the fitting numbers of *A*_1_, *A*_2_, and *A*_3_, shown in Figure 1 and Table 2. The measurements were performed under 0.01 s^−1^ shear rate, since *μ*_*p*_^0^ is the viscosity at very low shear rate.

*P*_*a*_ and *γ*_1/2_ are functions of *μ*_*p*_^0^ (or polymer concentration); the expressions are provided by CNPC based on their former research, shown in the following equations, respectively:

(3) $$\begin{array}{c} P_{a}=1.182{\left(\mu_{p}^{0}\right)}^{0.0341}, \end{array}$$

(4) $$\begin{array}{c} \gamma_{1/2}=376.2{\left(\mu_{p}^{0}\right)}^{-1.365}+0.0341. \end{array}$$

The relationship between effective shear rate *γ* and seepage velocity is shown in the following [36]:

(5) $$\begin{array}{c} \gamma =\frac{3n+1}{n+1}\frac{10^{4}v}{\sqrt{8C^{\prime }K\phi }}, \end{array}$$

(6) $$\begin{array}{c} v=\frac{Q}{2\pi rh}, \end{array}$$

where *n* is the bulk power law index, in the range of 0 to 1; *C*′ is tortuosity coefficient; *ϕ* is porosity; *K* is permeability; *Q* is flow rate of injected polymer solution; *h* is reservoir thickness; *r* is radial distance; *v* is Darcy velocity.

By considering IPV and permeability reduction caused by polymer flooding, (5) is changed to

(7) $$\begin{array}{c} \gamma =\frac{3n+1}{n+1}\frac{10^{4}v}{\sqrt{8C^{\prime }K_{p}\phi_{p}}}, \end{array}$$

where *K*_*p*_ is effective permeability, *K*_*p*_ = *K*/*R*_*k*_, *R*_*k*_ being permeability reduction coefficient; *ϕ*_*p*_ is effective porosity, *ϕ*_*p*_ = *ϕ*(1 − IPV).

During transport in porous medium, polymer concentration is also affected by convection and diffusion. Thus, polymer concentration by considering convection and diffusion is shown in the following [37]:

(8) $$\begin{array}{c} C_{p}\left(r,t\right)=\frac{C_{p0}}{2}-\frac{C_{p0}}{2}\mathrm{erf}\left[\frac{r-Vt}{2\sqrt{Dt}}\right], \end{array}$$

where *C*_*p*0_ is initial polymer concentration; *D* is diffusion coefficient.

There are several shear-thinning rheological models developed for polymer solutions. The model used in this study can accurately match the apparent viscosity of the proprietary HPAM polymer provided by CNPC over a wide range of injected velocity, especially when polymer solutions pass through the perforation.

## 3. Well Testing Modeling Methodology

**Figure 2:**
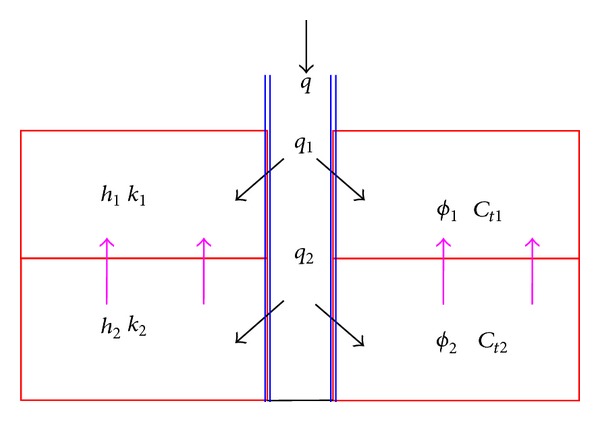
Sketch of polymer flooding in crossflow double-layer reservoir.

The percolation of polymer flooding in crossflow double-layer reservoir is sketched in Figure 2. Crossflow occurs in the interlayer and fluids can transport from low permeability zone to high permeability zone when polymer solutions are injected into the reservoir. The hypotheses are as follows: (1) polymer solutions and reservoir brines are miscible; (2) properties of polymer solutions are the same in each layer; (3) fluids flow satisfies Darcy's law; (4) each layer is homogeneous, but formation properties, for example, layer thickness, permeability, skin factor, and compressibility, are different between two layers; (5) gravity effect is negligible; (6) the initial pressure of each layer is the same, *p*_*i*_; (7) reservoir rocks and fluids are compressible; (8) process of polymer transportation is isothermal; (9) crossflow of interlayer is pseudosteady state.

Based on the rheological model and hypotheses discussed above, the well testing interpretation model in crossflow double-layer reservoir by polymer flooding is established, by considering shear, diffusion, convection, IPV, permeability reduction, wellbore storage effect, and different layered skin factors:

(i) percolation equation:
  (9) $$\begin{array}{c} K_{p1}h_{1}\frac{\partial }{\partial r}\left(r\frac{1}{\mu_{p}}\frac{\partial p_{1}}{\partial r}\right)+a\frac{K_{p2}h_{2}}{\mu_{p}}\left(p_{2}-p_{1}\right)=\phi_{p1}C_{t1}h_{1}\frac{\partial p_{1}}{\partial t}\\ K_{p2}h_{2}\frac{\partial }{\partial r}\left(r\frac{1}{\mu_{p}}\frac{\partial p_{2}}{\partial r}\right)-a\frac{K_{p2}h_{2}}{\mu_{p}}\left(p_{2}-p_{1}\right)=\phi_{p2}C_{t2}h_{2}\frac{\partial p_{2}}{\partial t}; \end{array}$$
(ii) internal boundary conditions:
  (10) wellbore  storage  effect   qB=Cdpwfdt−(Kp1h1μpr∂p1∂r+Kp2h2μpr∂p2∂r)|r=rwskin  factor  pw(t)=(p1−s1r∂p1∂r)|r=rw=(p2−s2r∂p2∂r)|r=rw;
(iii) external boundary condition (infinite boundary):
  (11) $$\begin{array}{c} p_{1}\left(\infty ,t\right)=p_{2}\left(\infty ,t\right)=p_{i}; \end{array}$$
(iv) initial condition:
  (12) $$\begin{array}{c} p_{1}\left(r,0\right)=p_{2}\left(r,0\right)=p_{i}, \end{array}$$
  where *p*_1_ and *p*_2_ are reservoir pressure of each layer; *K*_*p*1_ and *K*_*p*2_ are effective layered permeability; *h*_1_ and *h*_2_ are layer thickness; *C*_*t*1_ and *C*_*t*2_ are total layered compressibility; *ϕ*_*p*1_ and *ϕ*_*p*2_ are porosity; *C* is wellbore storage coefficient; *s*_1_ and *s*_2_ are skin factor; *p*_*wf*_ is BHP; *p*_*i*_ is initial reservoir pressure; *a* is flow-rate exchange coefficient.

Dimensionless parameters are involved after solving the model and obtaining BHP:

(13) $$\begin{array}{c} p_{wDj}=\frac{\sum_{j=1}^{2}{\left(k_{p}h\right)}_{j}}{1.842\times 10^{-3}q\mu_{p}B}\left(p_{wfj}-p_{i}\right),\;\left(j=1,2\right),\\ t_{D}=\frac{3.6\sum_{j=1}^{2}{\left(k_{p}h\right)}_{j}}{\sum_{j=1}^{2}{\left(\phi_{p}C_{t}h\right)}_{j}\mu_{p}r_{w}^{2}}t,\\ C_{D}=\frac{C}{2\pi r_{w}^{2}\sum_{j=1}^{2}{\left(\phi_{p}C_{t}h\right)}_{j}}, \end{array}$$

where *p*_*wD*_ is dimensionless BHP; *t*_*D*_ is dimensionless time; *C*_*D*_ is dimensionless wellbore storage coefficient; *μ*_*p*_ is the viscosity of the first grid, which expresses the rheology behavior of fluid near wellbore.

Three new parameters are then proposed in order to effectively analyze parameters sensitivity and interpret field test data:

(14) $$\begin{array}{c} \chi =\frac{K_{p1}h_{1}}{K_{p1}h_{1}+K_{p2}h_{2}},\\ \omega =\frac{{\phi_{p1}C}_{t1}h_{1}}{{\phi_{p1}C}_{t1}h_{1}+{\phi_{p2}C}_{t2}h_{2}},\\ \lambda =\frac{ar_{w}^{2}K_{p2}h_{2}}{K_{p1}h_{1}+K_{p2}h_{2}}, \end{array}$$

where *χ* is formation coefficient ratio; *ω* is storativity ratio; *λ* is interporosity flow coefficient.

## 4. Type Curves and Sensitivity Analysis

Based on dimensionless BHP and dimensionless BHP derivative, the type curves of pressure and pressure derivative in log-log scale are obtained. Sensitivity analysis is further investigated.

### 4.1. Type Curves

**Figure 3:**
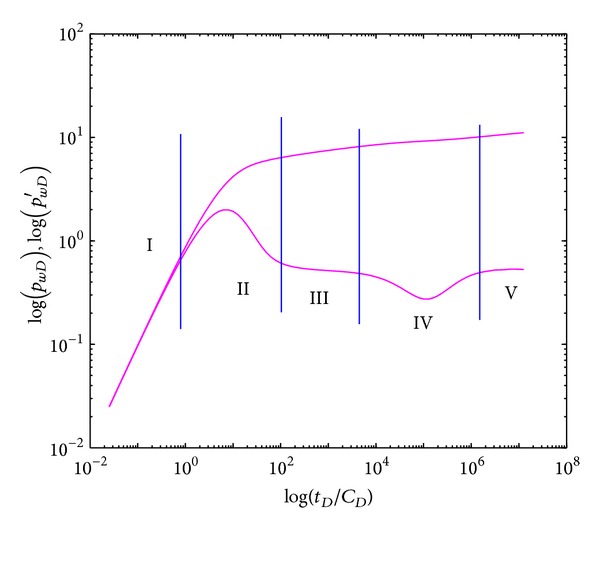
Type curves of well testing in crossflow double-layer reservoir by polymer flooding.

Type curves of well testing in crossflow double-layer reservoir by polymer flooding are shown in Figure 3, which shows that type curves have five flow segments: (I) wellbore storage section, where pressure and pressure derivative curves are superposed, reflecting the pressure response characteristics during well storage stage; (II) intermediate flow section (transient section), that describes the pressure response from pure wellbore storage stage to mid-radial flow stage within internal region, and there is a “convexity”; (III) mid-radial flow section, where fluids flow of individual layer achieves plane radial flow before crossflow happens, showing a horizontal period of pressure derivative line; (IV) crossflow section, where fluids in low permeability layer transport through interlayer into high permeability layer, and there is a “concave”; and (V) systematic radial flow section, where the whole system presents plane radial flow over time and the pressure curve lightly turns upward due to the influence of the non-Newtonian fluid properties of polymer solution.

**Figure 4:**
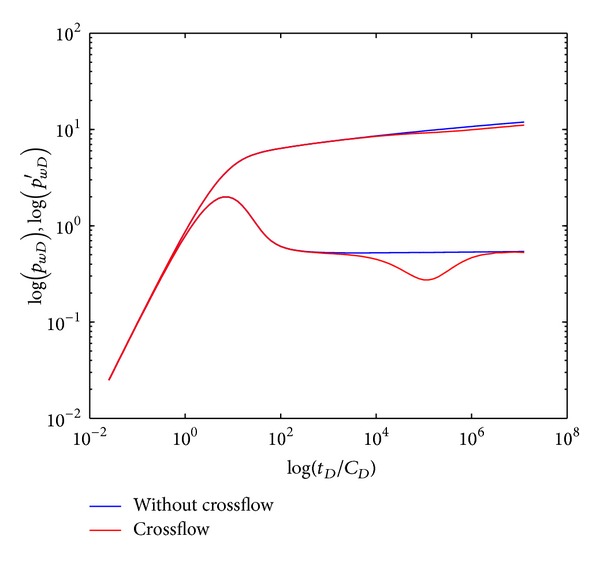
Type curves of well testing in double-layer reservoir by polymer flooding with and without crossflow.

The comparison of type curves in double-layer reservoir by polymer flooding with and without crossflow is demonstrated in Figure 4. It is obvious that there exists a “concave” (section IV) in the crossflow double-layer reservoir, which is formed by the fluids percolation from low permeability layer into high permeability layer resulting in crossflow through the interlayer. After crossflow is developed over time, the “concave” will vanish and curves will overlap when pressures of each layer achieve equilibrium. In systematic radial flow section (V), the BHP in crossflow reservoir is lower than that in noncrossflow reservoir since crossflow reduces the flow resistance (equal to systematic permeability enhanced); however, the BHP derivative is the same with value of 0.5.

### 4.2. Sensitivity Analysis

The effects of different parameters on type curves are investigated, including interporosity flow coefficient, ratio of formation coefficient, storativity ratio, initial polymer concentration, and IPV.

#### 4.2.1. Interporosity Flow Coefficient

**Figure 5:**
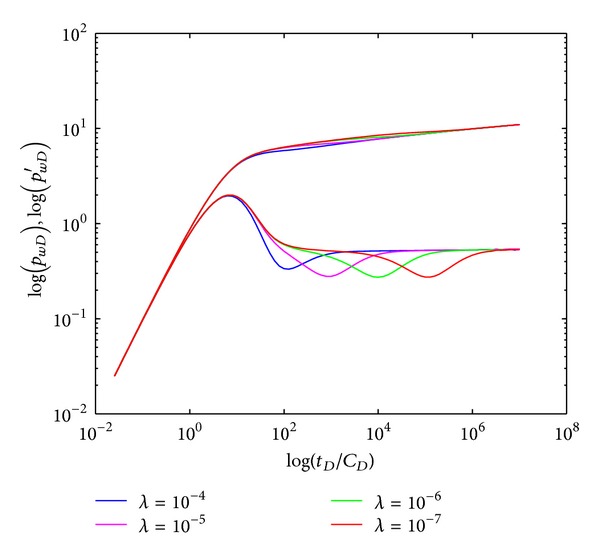
Effect of interporosity flow coefficient (*λ*) on type curves.

The influence of interporosity flow coefficient (*λ*) on type curves in crossflow double-layer reservoir by polymer flooding is shown in Figure 5. Smaller *λ* indicates fewer fluids transport through interlayer, which depends on the permeability difference and BHP difference between two layers. Smaller permeability difference or BHP difference results in small *a* and *λ*. The “concave” appears delayed with smaller interporosity flow coefficient since it needs more time for the fluids in crossflow section (IV) to achieve equilibrium. After that, individual layer reaches the plane radial flow and BHP derivative curve changes to horizontal, indicating fluids flow achieves systematic radial flow section (V). The time of “concave” appearance can qualitatively evaluate formation heterogeneity since it is influenced by layered permeability difference: it appears earlier in heterogeneous formation, and it appears later in relative homogenous formation.

#### 4.2.2. Formation Coefficient Ratio

**Figure 6:**
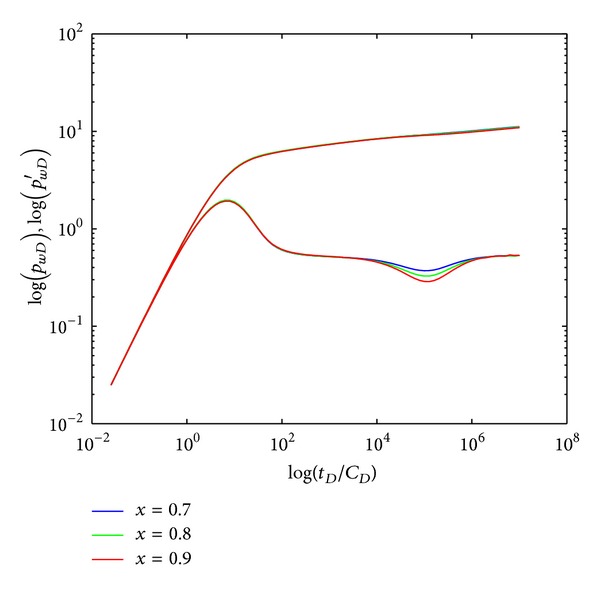
Effect of formation coefficient ratio (*χ*) on type curves.

Figure 6 represents the effect of formation coefficient ratio (*χ*) on type curves in crossflow double-layer reservoir by polymer flooding. It shows that *χ* only affects the crossflow section (IV): the smaller *χ*  is, the shallower the “concave” becomes and vice versa. For reservoirs with fixed value of layer permeability, smaller *χ* means smaller difference of layer thickness, and the “concave” becomes shallower as permeability difference decreases.

#### 4.2.3. Storativity Ratio

**Figure 7:**
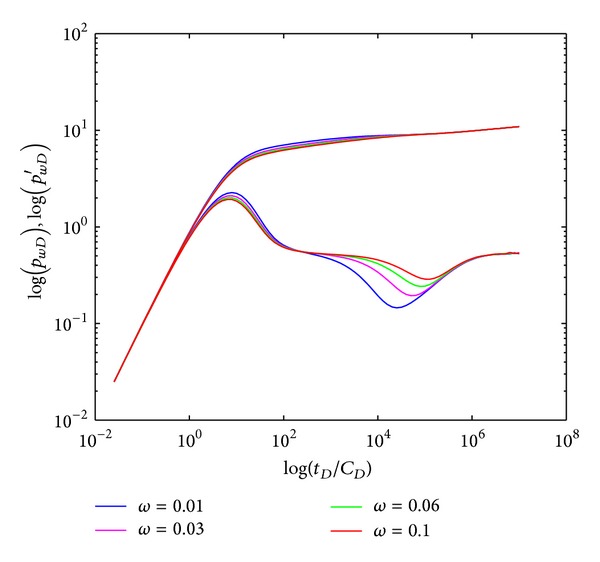
Effect of storativity ratio (*ω*) on type curves.

The effect of storativity ratio (*ω*) on type curves in crossflow double-layer reservoir by polymer flooding is shown in Figure 7. The width and depth of the “concave” are influenced by *ω*: the “concave” gradually becomes narrower and shallower when *ω* increases, and vice versa. Individual layers, respectively, reach their radial flow after the crossflow segment ends, indicating systematic radial flow section (V).

#### 4.2.4. Initial Polymer Concentration

**Figure 8:**
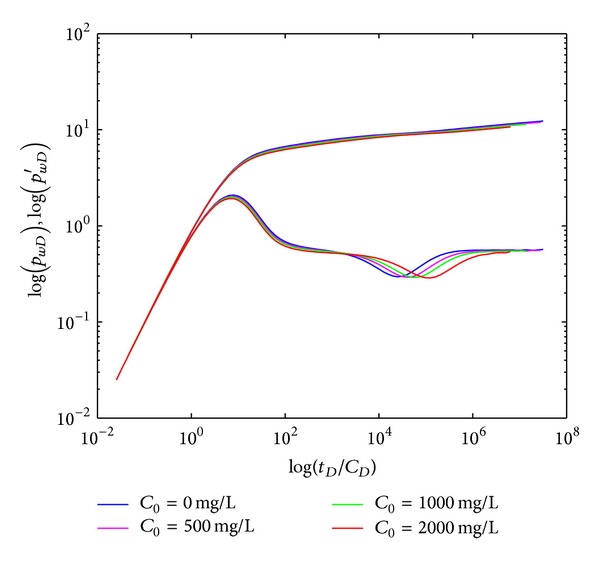
Effect of initial polymer concentration (*C*_*p*0_) on type curves.

The effect of initial polymer concentration (*C*_*p*0_) on type curves in crossflow double-layer reservoir by polymer flooding is shown in Figure 8, which indicate that the crossflow section (IV) appears later and BHP derivative curve in systematic radial flow section (V) turns more upward by increasing *C*_*p*0_. Since viscosity is increased for higher *C*_*p*0_, there is more flow resistance for fluids to transport through interlayer, resulting in delay of crossflow section (IV) appearance and greater amplitude of BHP derivative curve in systematic radial flow section (V). Consider *C*_0_ = 0 mg/L expressed as water flooding, which is Newtonian fluid with constant viscosity. Further investigation indicates that the effect of polymer rheology on type curve section (V) is dramatically reduced by crossflow, which means the pressure curve and pressure derivative curve of polymer flooding are similar to those of water flooding in section (V) and this phenomenon is also proved by field test data. However, the slope of type curves in one-layer reservoir with homogenous thickness by polymer flooding is much larger than that of water flooding.

#### 4.2.5. Inaccessible Pore Volume

**Figure 9:**
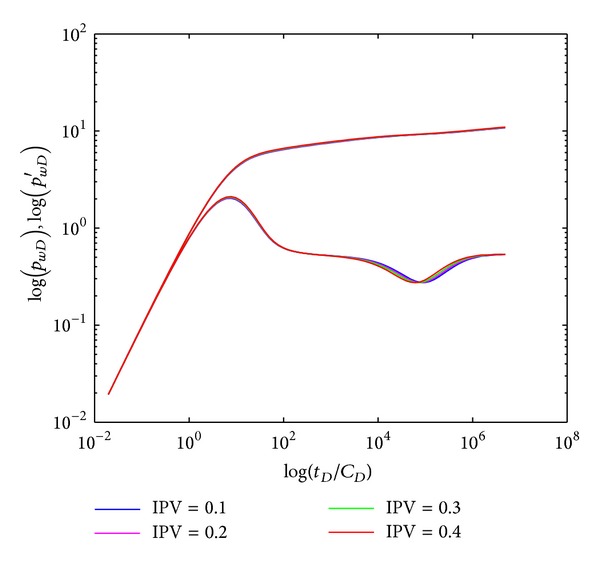
Effect of IPV on type curves.

Figure 9 represents the effect of IPV on type curves in crossflow double-layer reservoir by polymer flooding. The crossflow section (IV) appears earlier for reservoir with bigger IPV. Bigger IPV means lower effective porosity, and the fluid velocity is higher for the reservoir with fixed flow rate of polymer injected, resulting in earlier appearance of the crossflow section (IV) and systematic radial flow section (V). However, the effect of IPV on well testing type curves is unremarkable; moreover, the IPV caused by polymer flooding in oilfields is usually less than 0.15, so the effect of IPV can be negligible during well testing interpretation. Unlike other parameters, the effect of IPV on type curves is listed here only for theoretical analysis.

#### 4.2.6. Wellbore Storage Coefficient

**Figure 10:**
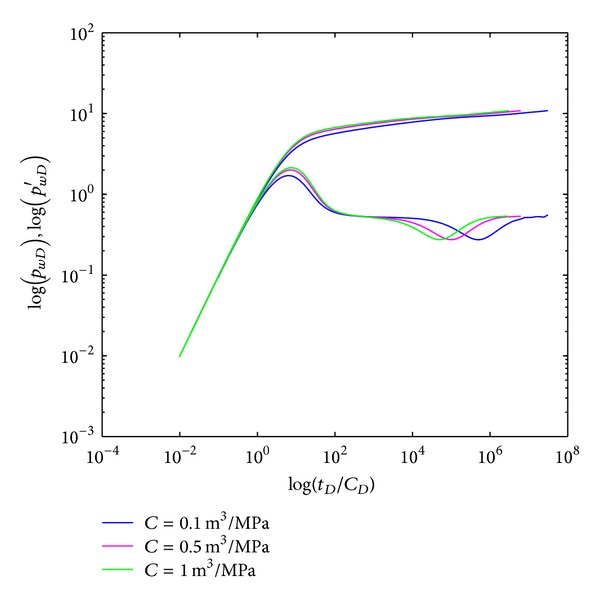
Effect of wellbore storage coefficient (*C*) on type curves.

The effect of wellbore storage coefficient on type curves in crossflow double-layer reservoir by polymer flooding is shown in Figure 10. The depth of the “concave” and “convexity” is influenced by *C*; however it does not affect the width. The crossflow section (IV) and systematic radial flow section (V) gradually appear earlier with *ω* increases; meanwhile, the mid-radial flow section (III) is shortened.

## 5. Field Tests Interpretation

Well testing data of field test was provided by CNPC. Then draw the BHP data with time in log-log scale. Interpret the data and perform history matching of type curves to evaluate reservoir formation and calculate the average formation pressure, layered permeability, layered skin factor, and wellbore storage coefficient. The interpretation results of layered permeability and layered skin factor are significant for oilfields, since oil industry will adjust development plan of production based on them. If the layered permeability is much lower or the layered skin factor is much higher than those of before polymer flooding, it indicates that polymer flooding leads to serious formation damage and specific methods should be employed to reduce formation damage and improve production, for example, acidizing.

### 5.1. Basic Properties of Oilfield

The tectonic surface area is 33 km^2^, and structure amplitude is about 100 m. The formation conditions and fluid properties are suitable for polymer flooding; meanwhile, relatively low salinity and low divalent cation concentration are beneficial to maintaining systematic viscoelasticity. The characteristics of crude oil under surface conditions and reservoir conditions are shown in Tables 3 and 4, respectively. The pressure derivative curve of field test data was modified for curve smoothing by using Bourdet's method [38].

### 5.2. Field Test One

Well testing was based on injection fall-off process. The polymer solutions were injected into double-layer reservoirs with initial concentration of 1600 mg/L, and the reservoir thickness is 14 m. Well 5-227 performed polymer flooding from Feb 1, 2012, to May 7, 2012, and then the polymer injection was stopped and pressures were measured. It took three days for well testing, and polymer flooding was performed again since May 10, 2012. Basic parameters of well and reservoir are shown in Table 5.

**Figure 11:**
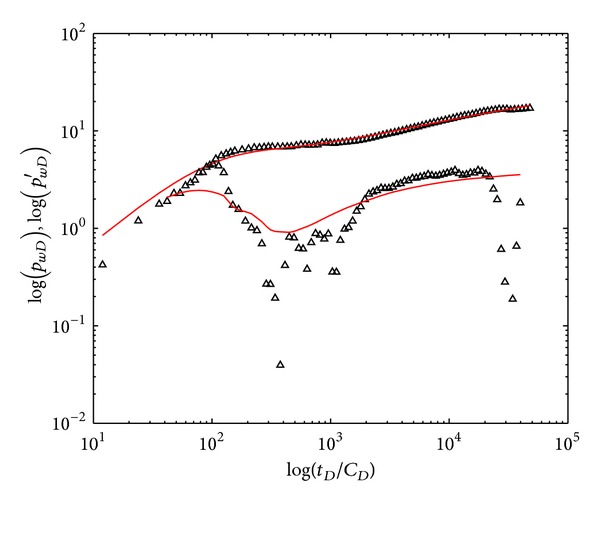
Field test data and history matching of type curves (Field Test One: Well 5-227).

The history matching curves and field testing data are shown in Figure 11, and the interpretation results are shown in Table 6. The permeability and skin factor of individual layer acquired by interpreting field test data are consistent with the actual situation of oilfield, indicating that our model can accurately interpret Field Test One and evaluate formation. Meanwhile, polymer flooding results in negligible permeability reduction or formation damage in this case, since the interpreted permeability and skin factors are nearly the same as those of before polymer flooding.

### 5.3. Field Test Two

Well testing was also based on injection fall-off process. The polymer solutions were injected into double-layer reservoirs with initial concentration of 1600 mg/L, and the reservoir thickness is 21 m. Well 5-225 (500 meters away from Well 5-227) performed polymer flooding from Feb 1, 2012, to Apr. 28, 2012, and then the polymer injection was stopped and pressures were measured (nine days before Field Test One). It took three days for well testing, and polymer flooding was performed again since May 1, 2012. Basic parameters of well and reservoir are shown in Table 7.

**Figure 12:**
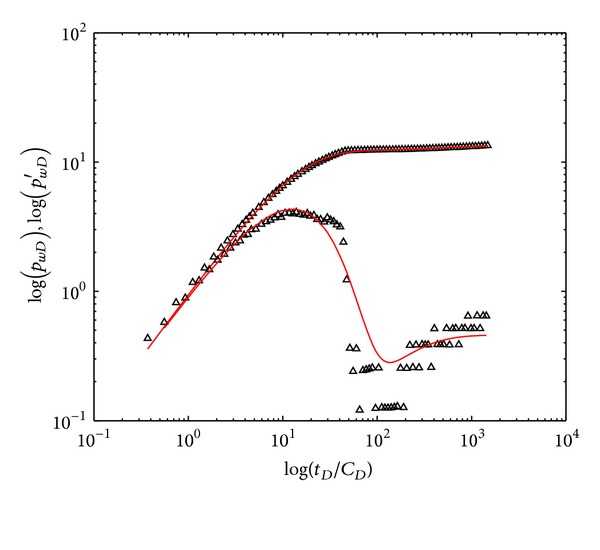
Field test data and history matching of type curves (Field Test Two: Well 5-225).

The history matching curves and field testing data are shown in Figure 12, and the interpretation results are shown in Table 8. The occurrence of “concave” is earlier than Field Test One, due to the bigger permeability difference between two layers. The skin factor of individual layer and layer 1 permeability acquired by interpreting field test data are consistent with the actual situation of oilfield, which further prove that our model can accurately interpret Field Test Two and evaluate formation. Moreover, the layer 2 permeability is 68 mD and permeability reduction coefficient is 3.1 on average, indicating formation was damaged by polymer flooding. Blockage removal agent was further injected into the reservoir and layer 2 permeability was increased to 174 mD, resulting in 2.4% EOR of individual well.

## 6. Conclusion

This work established well testing models for crossflow double-layer reservoirs by polymer flooding. Type curves of numerical well testing were obtained, and field test data were further interpreted and history-matched. The main conclusions drawn from this study are as follows.

1. The model developed in this work by considering IPV, permeability reduction, shear rate, diffusion, and convection can accurately demonstrate rheological behavior of the proprietary HPAM polymer provided by CNPC over a wide range of injected velocity, especially when polymer solutions pass through the perforation.
2. Type curves have five sections with different flow status: (I) wellbore storage section, where pressure and pressure derivative curves are superposed, reflecting the pressure response characteristics during well storage stage; (II) intermediate flow section (transient section between wellbore storage section and mid-radial flow section); (III) mid-radial flow section, where fluids flow of each layer achieves plane radial flow before crossflow occurs; (IV) crossflow section where fluids in low permeability layer transport through interlayer into high permeability layer; and (V) systematic radial flow section, where the whole system presents plane radial flow over time.
3. The remarkable feature of the crossflow in type curves is the occurrence of “concave.” The effect of polymer rheology on type curve section (V) is dramatically reduced by crossflow, which means the pressure curve and pressure derivative curve of polymer flooding are similar to those of water flooding in systematic radial flow section (V). Sensitivity analysis was performed to investigate the effect of different parameters on the type curves, including interporosity flow coefficient, formation coefficient ratio, storativity ratio, initial polymer concentration, IPV, and wellbore storage coefficient. The influence of IPV on the well testing in polymer flooding reservoirs can be neglected, since polymer flooding usually results in unremarkable IPV.
4. Field tests were conducted in two wells of crossflow double-layer reservoirs by polymer flooding. The field test data were interpreted and history-matched by employing our well testing interpretation method, which indicated our model can accurately interpret field test data and evaluate formation. Moreover, formation damage caused by polymer flooding can also be evaluated by comparison of the interpreted permeability with initial layered permeability before polymer flooding. If interpreted permeability is much lower than initial permeability, specific techniques should be employed to eliminate formation damage and enhance oil recovery.

## Figures and Tables

**Table 1:** Synthetic brine composition.

Total salinity	NaCl	MgCl_2_	CaCl_2_	Na_2_SO_4_
4.3 wt%	3.44 wt%	0.18 wt%	0.64 wt%	0.04 wt%

**Table 2:** Characteristics of polymer solutions.

*μ*_*w*_, (mPa*·*s)	*A*_1_, (g/L)^−1^	*A*_2_, (g/L)^−2^	*A*_3_, (g/L)^−3^	*C*_*p*0_, (g/L)	*D*, (cm^2^/s)
0.5	0.642	0.201	0.931	1.750	0.0246

**Table 3:** Characteristics of crude oil under surface conditions.

Density (g/cm^3^, 20°C)	Viscosity (mPa*·*s, 20°C)	Viscosity (mPa*·*s, 55°C)
0.925~0.934	407.5~533.6	49.56~58.21

**Table 4:** Characteristics of crude oil under reservoir conditions.

Density (g/cm^3^)	Viscosity (mPa*·*s)	Volume factor	Saturation pressure (MPa)	Oil-gas ratio	Acid number
0.8675	14.2	1.1038	12.70	42	0.4~1.16

**Table 5:** Basic parameters of well and reservoir for 5-227 field test.

Injection rate	*q* (m^3^/d)	100
Layer 1 thickness	*h*_1_ (m)	8
Layer 2 thickness	*h*_2_ (m)	6
Oil volume factor	*B*_0_	1.1037
Porosity	*ϕ*	0.3
Crude oil viscosity	*μ*_*o*_ (mPa*·*s)	14.2
Brine viscosity	*μ*_*w*_ (mPa*·*s)	0.5
Temperature	°C	75
Total compressibility	*C*_*t*_ (1/MPa)	0.0014
Well radius	*r*_*w*_ (m)	0.1
Layer 1 permeability before polymer flooding	mD	1592
Layer 2 permeability before polymer flooding	mD	1466
Layer 1 skin factor before polymer flooding	n/a	1.11
Layer 2 skin factor before polymer flooding	n/a	1.18

**Table 6:** Interpretation results of Field Test One (Well 5-227).

Average reservoir pressure	MPa	17.26
Layer 1 permeability	mD	1570
Layer 2 permeability	mD	1460
Layer 1 skin factor	n/a	1.13
Layer 2 skin factor	n/a	1.20
Wellbore storage coefficient	m^3^/MPa	0.60

**Table 7:** Basic parameters of well and reservoir for 5-225 field test.

Injection rate	*q* (m^3^/d)	136
Layer 1 thickness	*h*_1_ (m)	12
Layer 2 thickness	*h*_2_ (m)	9
Oil volume factor	*B*_0_	1.1037
Porosity	*ϕ*	0.25
Crude oil viscosity	*μ*_*o*_ (mPa*·*s)	14.2
Brine viscosity	*μ*_*w*_ (mPa*·*s)	0.5
Temperature	°C	75
Total compressibility	*C*_*t*_ (1/MPa)	0.0014
Well radius	*r*_*w*_ (m)	0.1
Layer 1 permeability before polymer flooding	mD	1352
Layer 2 permeability before polymer flooding	mD	211
Layer 1 skin factor before polymer flooding	n/a	2.49
Layer 2 skin factor before polymer flooding	n/a	0.37

**Table 8:** Interpretation results of Field Test Two (Well 5-225).

Average reservoir pressure	MPa	18.56
Layer 1 permeability	mD	1340
Layer 2 permeability	mD	68
Layer 1 skin factor	n/a	2.56
Layer 2 skin factor	n/a	1.98
Wellbore storage coefficient	m^3^/MPa	0.54

## Annotation

ASP: Alkali-surfactant-polymer
*C*: Wellbore storage coefficient
*C*_*p*0_: Initial polymer concentration
*C*_*D*_: Dimensionless wellbore storage coefficient
*C*_*p*_: Polymer concentration
*C*′: Tortuosity coefficient
*C*_*t*_, *C*_*t*1_, *C*_*t*2_: Total compressibility
*D*: Diffusion coefficient
EOR: Enhanced oil recovery
HPAM: Hydrolyzed polyacrylamide
IPV: Inaccessible pore volume
*K*: Permeability
*K*_*p*_, *K*_*p*1_, *K*_*p*2_: Effective permeability
*p*, *p*_1_, *p*_2_: Reservoir pressure
*p*_*i*_: Initial reservoir pressure
*p*_*wD*_: Dimensionless BHP
*Q*: Flow rate
*R*_*k*_: Permeability reduction coefficient
*a*: Flow-rate exchange coefficient
*h*, *h*_1_, *h*_2_: Reservoir thickness
*n*: Bulk power law index
*P*_*a*_, *A*_1_, *A*_2_, *A*_3_, *C*_SEP_^SP^: Fitting parameters
*p*_*wf*_ BHP: Bottom hole pressure
*r*: Radial distance
*s*_1_, *s*_2_: Skin factor
*t*_*D*_: Dimensionless time
*ϕ*_*p*_, *ϕ*_*p*1_, *ϕ*_*p*2_: Effective porosity
*χ*: Formation coefficient ratio
*γ*: Effective shear rate
*γ*_1/2_: Shear rate at which apparent viscosity is the average of *μ*_*∞*_ and *μ*_*p*_^0^
*λ*: Interporosity flow coefficient
*μ*_*p*_: Apparent viscosity of polymer solution
*μ*_*p*_^0^: Viscosity of polymer solution at low shear rate
*μ*_*w*_: Brine viscosity
*μ*_*∞*_: Viscosity of polymer solution at infinite shear rate
*v*: Darcy velocity
*ϕ*: Porosity
*ω*: Storativity ratio.

## References

[1] Jamaloei BY, Kharrat R, Torabi F (2010). Analysis and correlations of viscous fingering in low-tension polymer flooding in heavy oil reservoirs. *Energy and Fuels*.

[2] Bera A, Mandal A, Guha BB (2014). Synergistic effect of surfactant and salt mixture on interfacial tension reduction between crude oil and water in enhanced oil recovery. *Journal of Chemical & Engineering Data*.

[3] Farajzadeh R, Ameri A, Faber MJ, Batenburg DW, Boersma DW, Bruining J (2012). Effect of continuous, trapped, and flowing gas on performance of Alkaline Surfactant Polymer (ASP) flooding. *Industrial & Engineering Chemistry Research*.

[4] Yu H, Kotsmar C, Yoon KY Transport and retention of aqueous dispersions of paramagnetic nanoparticles in reservoir rocks.

[5] Zhang T, Murphy M, Yu H Investigation of nanoparticle adsorption during transport in porous media.

[6] Shiran BS, Skauge A (2013). Enhanced oil recovery (EOR) by combined low salinity water/polymer flooding. *Energy and Fuels*.

[7] Ren G, Zhang H, Nguyen QP (2011). Effect of surfactant partitioning between CO_2_ and water on CO_2_ mobility control in hydrocarbon reservoirs. *SPE Paper*.

[8] Ren G, Sanders AW, Nguyen QP (2014). New method for the determination of surfactant solubility and partitioning between CO_2_ and brine. *The Journal of Supercritical Fluids*.

[9] Wyatt NB, Gunther CM, Liberatore MW (2011). Increasing viscosity in entangled polyelectrolyte solutions by the addition of salt. *Polymer*.

[10] Lake LW (1996). *Enhanced Oil Recovery*.

[11] Lefkovits HC, Hazebroek P, Allen EE, Matthews CS (1961). A study of the behaviour of bounded reservoir composed of stratified layers. *Society of Petroleum Engineers Journal*.

[12] Tariq SM, Ramey HJ (1978). Drawdown behavior of a well with storage and skin effect communicating with layers of different Radii and other characteristics. *SPE Paper*.

[13] Kucuk F, Karakas M, Ayestaran L (1986). Well testing and analysis techniques for layered reservoirs. *SPE Formation Evaluation*.

[14] Kuchuk FJ, Shah PC, Ayestaran L, Nicholson B Application of multilayer testing and analysis: a field case.

[15] Bourdet D, Johnston F (1985). pressure behavior of layered reservoirs with crossflow. *SPE paper*.

[16] Ehlig-Economides CA, Joseph J (1987). A new test for determination of individual layer properties in a multilayered reservoir. *SPE Formation Evaluation*.

[17] Gao C-T (1984). Single phase fluid flow in a stratified porous medium with crossflow. *Society of Petroleum Engineers Journal*.

[18] Jackson RR, Banerjee R, Thambynayagam RKM An integrated approach to interval pressure transient test analysis using analytical and numerical methods.

[19] Kamal MM, Pan Y, Landa JL, Thomas OO Numerical well testing: a method to use transient testing results in reservoir simulation.

[20] Mijinyawa A, Alamina P, Orekoya A (2010). An integrated approach to well test analysis-use of numerical simulation for complex reservoir systems. *SPE paper*.

[21] Zhang L, Guo J, Liu Q (2010). A well test model for stress-sensitive and heterogeneous reservoirs with non-uniform thicknesses. *Petroleum Science*.

[22] Veerabhadrappa SK, Trivedi JJ, Kuru E (2013). Visual confirmation of the elasticity dependence of unstable secondary polymer floods. *Industrial and Engineering Chemistry Research*.

[23] Zhang H, Challa RS, Bai B, Tang X, Wang J (2010). Using screening test results to predict the effective viscosity of swollen superabsorbent polymer particles extrusion through an open fracture. *Industrial and Engineering Chemistry Research*.

[24] Hoek PVD, Mahani H, Sorop T Application of injection fall-off analysis in polymer flooding.

[25] Jian J, Hou Q, Cheng L, Liu W, Li J, Zhu Y Recent progress and effects analysis of surfactant-polymer flooding field tests in China.

[26] Liu J, Guo Y, Hu J (2012). Displacement characters of combination flooding systems consisting of gemini-nonionic mixed surfactant and hydrophobically associating polyacrylamide for bohai offshoreoilfield. *Energy and Fuels*.

[27] Shi J, Varavei A, Huh C, Delshad M, Sepehrnoori K, Li X (2011). Transport model implementation and simulation of microgel processes for conformance and mobility control purposes. *Energy & Fuels*.

[28] Ali L, Barrufet MA (1994). Profile modification due to polymer adsorption in reservoir rocks. *Energy & Fuels*.

[29] Lai N, Qin X, Ye Z, Li C, Chen K, Zhang Y (2013). The study on permeability reduction performance of a hyperbranched polymer in high permeability porous medium. *Journal of Petroleum Science and Engineering*.

[30] Ogunberu AL, Asghari K (2004). *Water Permeability Reduction under Flow-Induced Polymer Adsorption*.

[31] Seright RS (2009). Disproportionate permeability reduction with pore-filling gels. *SPE Journal*.

[32] Bondor PL, Hirasaki GJ, Tham MJ (1972). Mathematical simulation of polymer flooding in complex reservoirs. *Society of Petroleum Engineers Journal*.

[33] Carreau PJ (1968). *Rheological equations for molecular network theories [PhD dissertation]*.

[34] Mete DM, Bird RB (1964). Tube flow of non-newtonian polymer solutions: part I. Laminar flow and rheological model. *AIChE Journal*.

[35] Flory PG (1953). *Principles of Polymer Chemistry*.

[36] Wang X (1990). Determination of the main parameters in the numerical simulation of polymer flooding. *Petrol Exploration Development*.

[37] Wang J (2008). *Physic-Chemical Fluid Mechanics and Application in Chemical EOR*.

[38] Bourdet D, Ayoub JA, Pirard YM (1989). Use of pressure derivative in well test interpretation. *SPE Journal*.

